# When gravity is not where it should be: How perceived orientation affects visual self-motion processing

**DOI:** 10.1371/journal.pone.0243381

**Published:** 2021-01-06

**Authors:** Meaghan McManus, Laurence R. Harris

**Affiliations:** Centre for Vision Research, York University, Toronto, ON, Canada; Johns Hopkins University, UNITED STATES

## Abstract

Human perception is based on expectations. We expect visual upright and gravity upright, sensed through vision, vestibular and other sensory systems, to agree. Equally, we expect that visual and vestibular information about self-motion will correspond. What happens when these assumptions are violated? Tilting a person from upright so that gravity is not where it should be impacts both visually induced self-motion (vection) and the perception of upright. How might the two be connected? Using virtual reality, we varied the strength of visual orientation cues, and hence the probability of participants experiencing a visual reorientation illusion (VRI) in which visual cues to orientation dominate gravity, using an oriented corridor and a starfield while also varying head-on-trunk orientation and body posture. The effectiveness of the optic flow in simulating self-motion was assessed by how much visual motion was required to evoke the perception that the participant had reached the position of a previously presented target. VRI was assessed by questionnaire When participants reported higher levels of VRI they also required less visual motion to evoke the sense of traveling through a given distance, regardless of head or body posture, or the type of visual environment. We conclude that experiencing a VRI, in which visual-vestibular conflict is resolved and the direction of upright is reinterpreted, affects the effectiveness of optic flow at simulating motion through the environment. Therefore, any apparent effect of head or body posture or type of environment are largely indirect effects related instead, to the level of VRI experienced by the observer. We discuss potential mechanisms for this such as reinterpreting gravity information or altering the weighting of orientation cues.

## Introduction

As we move about the world our senses keep track of our body’s location and orientation in space. One of these senses is our vestibular system, which helps determine our head’s orientation and movement. Within our vestibular system are the otoliths, which indicate linear acceleration. Tilting the head backwards or forwards relative to gravity displaces the hairs on the macula of the utricle of the otolith system in the same way as forward or backwards acceleration, respectively. This tilt-translation ambiguity poses a challenge to the brain to resolve the otolith’s signal into components due to gravity and to self-motion. One way it might be resolved is to compare the otolith’s signal with other information such as visual [1] or somatosensory cues (such as pressure on the feet while standing) [2, 3]. Additionally, motion sensitive neurons in the brainstem and cerebellum (with information provided by the otoliths and semicircular canals) may also help to disambiguate tilt and translation by using their patterns of activity to construct an internal model of our movement [4]. Additionally, there is also an assumption, or prior, that the top of the head is “up” [5] which may also contribute.

Signals from the semicircular canals, which are part of the vestibular organ, can be used to help disambiguate tilt and translation [6, 7]. The activity of Purkinje cells, in the cerebellar cortex, is modulated by semicircular canal activation when head orientation changes. This information then modulates the otolith acceleration signal to extract the translation [8]. However, at low frequency, or static head tilts, the semicircular canal signals cannot help disambiguate tilt/translation [8–10] and a linear acceleration may be interpreted as a tilt [8]. For instance, when pilots accelerate, the plane’s acceleration can be misinterpreted as tilt: a perception known as the somatogravic illusion [1, 11]. The presence of visual information however can affect how this signal is interpreted, where a view of the horizon and the visible relative motion of the ground, can reduce the occurrence of the illusion [1] by disambiguating the vestibular cue leading to a correct perception of translation rather than tilt. Such observations illustrate the interaction between visual and vestibular cues that is necessary to interpret self-motion and orientation cues. Here, we investigate how visual cues to orientation such as a view of the horizon, might affect the perception of visually induced self-motion.

In microgravity environments, astronauts’ perceived orientation is necessarily based exclusively on visual and body cues. Astronauts might orient themselves by regarding the area below their feet as the floor, or by using landmarks. However, when faced with a polarized object such as a coworker in a different orientation, or when moving into a new cabin, astronauts can experience a shift in their perceived orientation in which they reinterpret where they perceive up to be. This reinterpretation of “up”—based on visual cues—is called a visual reorientation illusion (VRI) [12].

**Fig 1:**
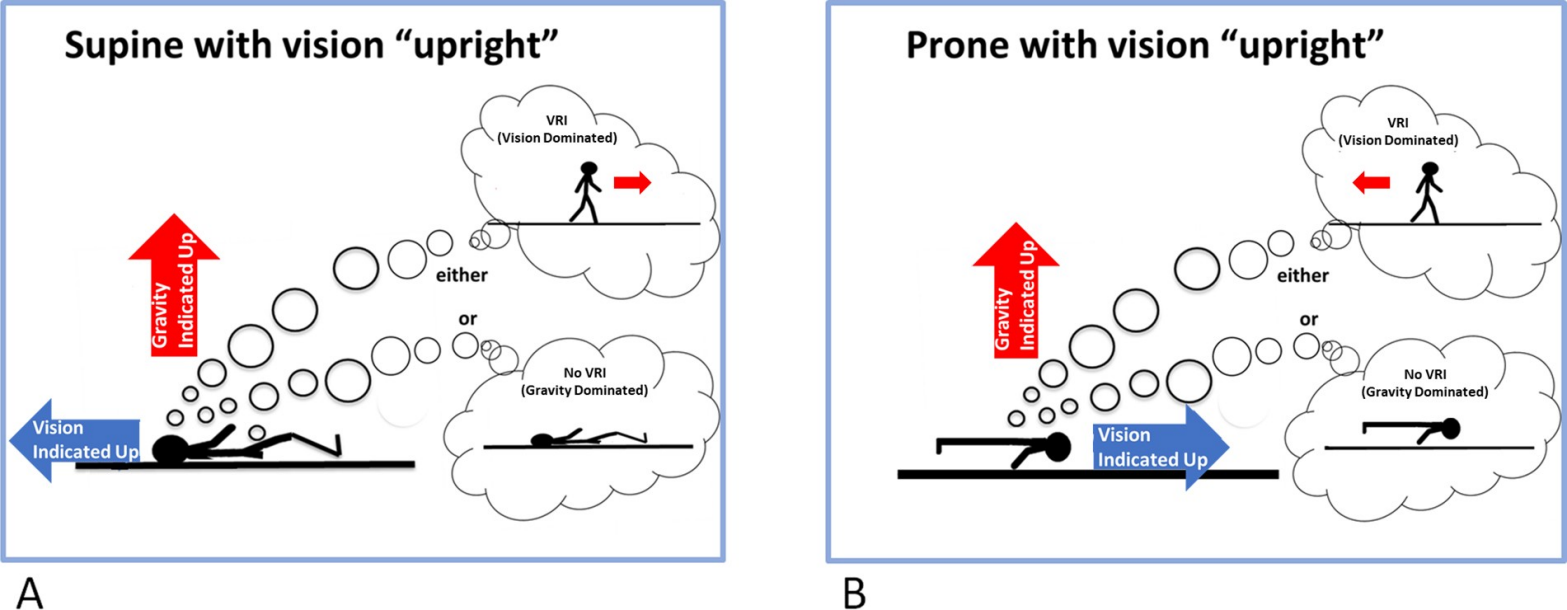
A visual reorientation illusion. A person lying down in an environment that has visual cues indicating one upright (blue arrow) and gravity cues indicating another upright (red arrow). The arrows point in the direction of up signaled by each cue. In (A) the person is supine and in (B) they are prone. If the visually-indicated upright were to dominate, the person would experience a visual reorientation illusion (VRI) and perceive themselves as upright (top thought clouds). If gravity cues were to dominate, they would perceive themselves supine or prone (bottom thought clouds).

VRI’s can also be experienced on earth in a specially constructed tilted room, using a mirror bed [13] or in virtual reality. If a person lies supine in a 90-degree-tilted room, such that the polarity of the objects and decorations of the room indicate that they are actually upright, 84% of people will experience a VRI and feel that they are upright [14] even though gravity is providing cues that they are horizontal (Fig 1). A mirror bed, with a cleverly arranged mirror at 45°, achieves the same thing [13].

If a person were tilted but believed that they were upright–that is, they were experiencing a VRI—how might they interpret the still-present vestibular and somatosensory cues consistent with a different body orientation? If the person were to correctly perceive themselves as supine, they should simply interpret the vestibular signal as confirming this. However, with a VRI the person appears to not be interpreting the vestibular cue as indicating tilt. One possibility is that under some conditions, such as in an environment with an ambiguous up where a tilted participant perceives themselves as standing, the vestibular acceleration cue provided by gravity might be at least partially reinterpreted as translation (Fig 1, top thought clouds). Crane [15] found that tilting a person’s head and body by 10 degrees forwards or backwards affected how they perceived a physical motion stimulus. The participants in Crane’s [15] study were seated on a moving platform and were moved in the fore-aft direction, with a control condition in which there was no movement. The participants had to indicate in which direction they thought they had moved. When a participant’s head and body were tilted back and they were translated at a velocity of 1.4 ± 0.7 cm/s backwards, they perceived themselves as stationary. Similarly, when tilted forwards and translating at 1.9 ± 0.5cm/s forwards they again interpreted the motion as corresponding to them being static. Then, when participants were tilted backward, they perceived a static condition as forwards motion and vice versa when tilted forward. These results suggest a possible misinterpretation of the gravity cue while tilted as indicating translation. However, tilting only the head did not bias their perception of motion. Crane [15] suggested that the neck muscles that were activated during head tilt might help to minimize any tilt-translation confusion so that only when the head and body were tilted together would the person interpret the vestibular signal as translation.

When upright, seeing visual cues consistent with forwards self-motion can be enough to evoke the sensation of self-motion, a phenomenon known as vection [16, 17] even though the vestibular signal indicates no physical acceleration. People are able to use the visual cues alone to update their sense of location as they experience simulated motion through an environment [18–21]. However, while supine and viewing visual motion consistent with forwards self-motion (relative to the body), the acceleration of gravity is now in a direction aligned with the visual motion. If gravity were to be interpreted as a physical acceleration, we might expect an enhancement of the visual motion cues in simulating the experience of movement through the environment, in which a given amount of visual motion might make them feel they were going faster and further (Fig 1A). Similarly, while prone the acceleration of gravity would oppose the visual motion which might make them feel they had travelled less far (Fig 1B). We will refer to this as the “additive hypothesis”. Previous studies have found conflicting evidence as to whether tilting a person affects the perception of vection [22–25]. It appears that changing body orientation (upright, prone, supine relative to gravity) and the direction of visual motion (forwards, backwards, upwards or downwards relative to the viewer) impacts the interpretation of motion that depends on which aspect of vection is being measured (e.g., onset, duration, or magnitude), but even for a single metric, results differ across experiments. For instance, the time to the onset of the sensation of vection increases while standing compared to prone for forward linear motion [22], but is reduced while standing relative to when supine [24], although others have found no difference in vection onset latencies between standing and supine [25]. The duration of the sensation of vection also varies depending on both body posture and the direction of visual motion. Vection duration increases the further the body tilts from upright but only during vertical visual motion with no change for vection duration with body tilt during horizontal visual motion during body tilt [23]. Lastly, the magnitude of the sensation of vection also appears to be affected by body posture where it is weaker when prone compared to sitting, with no difference in magnitude found between supine and sitting or supine and prone for forward vection [22], although others have found no differences in magnitude dependent on posture [24].

Additionally, cognitive factors such as the perceived context of the visual motion might influence the interpretation of self-motion (see review by [26]). Certain cognitive factors that might result in the weakening of the experience of vection include exposure to information suggesting that the observer is stationary, such as when the feet are touching a floor surface compared to when free floating in microgravity [27], when the ability to walk is inhibited such as while wearing iron shoes compared to wooden shoes [28], when participants have previous exposure to walking without experiencing optic flow [29], and when attentional load is increased during vection [30]. Additionally, Guterman and Allison [31] found that vertical vection in an environment made of tubular pipes enhanced vection compared to when participants were in a bubble environment, but only if participants were upright: no difference in vection was found when the participants were tilted. The authors state that during debriefing their participants reported that while upright in the pipe environment they felt as if they were in an elevator but did not experience this while lying down or in the bubble environment. This suggests that it was not posture per se that led to the changes in vection experience, as we should then see changes in vection in both postures while standing compared to lying, but instead the participants’ “experience” of the environment which lead to changes in vection. We will refer to this as the “cognitive hypothesis” which makes no explicit prediction about the direction of the effect of having a VRI on the effectiveness of visual motion, merely that an effect will occur. Our cognitive hypothesis does not rely on a misinterpretation of tilt as translation, but instead hypothesizes that differences will occur depending on how participants interpret their visual environment. For instance, a participant who is tilted prone and viewing optic flow towards their face simulating gravitationally downward movement, perceives themselves as falling. If the participant instead perceived themselves as upright, they may reinterpret their motion as forwards motion. It is possible that a participant who views their self-motion as “falling” may experience vection differently than someone who cognitively interprets that they are moving forwards. Guterman and Allison’s [31] study was unusual in asking their participants about their perceived experience. Most previous studies have not allowed for the possibility that participants may be interpreting the presented visual environments differently. This cognitive approach could help to explain some of the conflicting findings in the literature described above [22–25].

**Fig 2:**
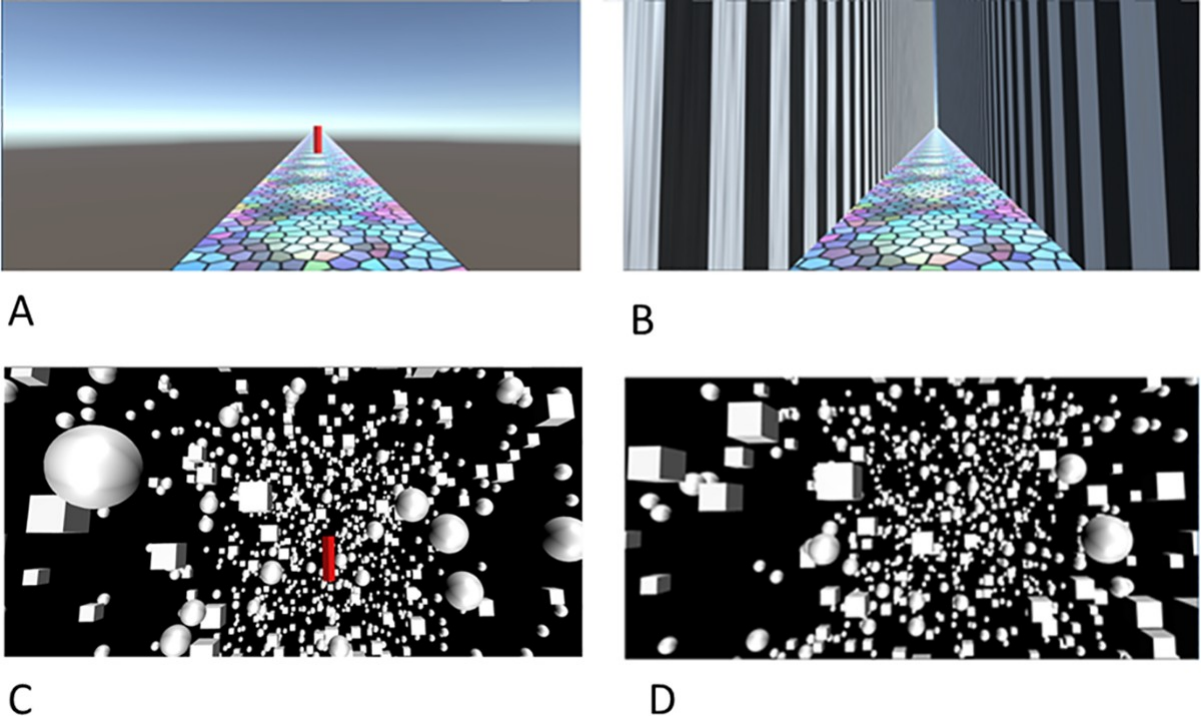
The virtual environments. Screen captures of the hallway environment (A and B) and the starfield environment (C and D). A and C are what the scenes looked like before movement while the target (vertical red line) was visible and the walls were invisible, B and D are what the scenes looked like once the participant had clicked the left mouse button and the optic flow started to simulate motion down the hallway. The hallway environment was used in Experiments 1, 2, and 3, the starfield environment was used only in Experiment 3.

In order to address these issues, we manipulated people’s physical and perceived body orientation and compared the effectiveness of optic flow (visual motion) at simulating motion through an environment in three separate experiments using two visual environments (see Fig 2). In Experiment 1 participants were standing, or tilted supine or prone, and were placed in a virtual reality environment with multiple polarized cues indicating that upright was always towards the top of the head (Fig 2A and 2B). We expected this environment to induce a VRI while tilted. We hypothesized that a participant experiencing a VRI may then at least partially reinterpret gravity as linear translation. Within this virtual world, participants were visually accelerated at 9.8m/s^2^ (1g) forwards. The visual motion profile was chosen to match the gravitational cue in magnitude to make the visual and gravitational cues more likely to be confused and thus to increase the likelihood that participants might misinterpret the vestibular cue as visual motion. Additionally, the use of static tilt could also help to lead to errors in disambiguating tilt from translation [8] with the visual environment supporting an interpretation of being upright and translating, similar to a reverse somatogravic illusion.

**Fig 3:**
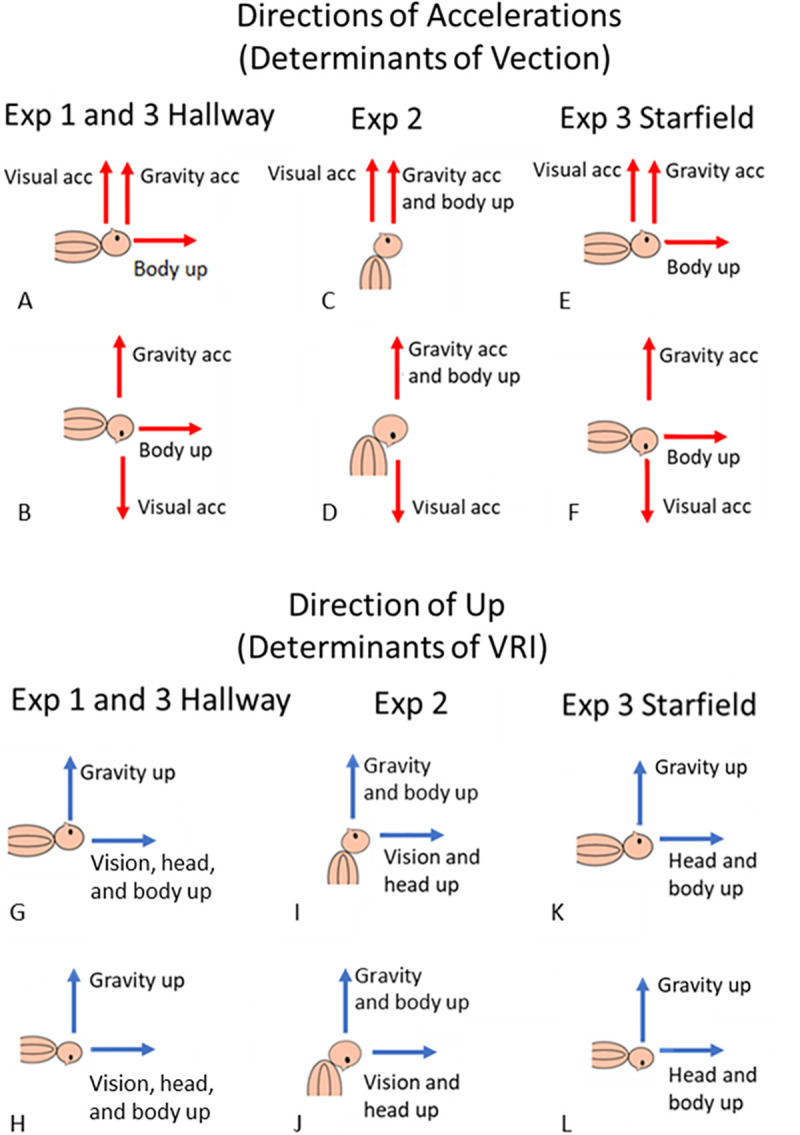
Gravity, vision, and perceived up by posture. The figure compares how gravity and vision (top panel) and perceived up (bottom panel) are affected by body and head posture. The red arrows in the top panel (A-F) show the visual and gravitational accelerations experienced by the participant in the same body postures as in the bottom panel G-L. When looking up (A, C, E) the direction of the visually simulated self-motion is in the same direction as the reaction to the gravitational acceleration; when looking down they are opposed (B, D, F). The bottom panel G-L shows the contributions to the cues to upright (blue arrows) while wearing a VR HMD with visual cues consistent with “up” being towards the top of the head (see Fig 2A and 2B), and with the head, or head and body tilted. In the starfield environment (see Fig 2C and 2D) there is no “visual up” (K, L).

If participants who had a VRI partially reinterpreted gravity as linear translation then, when the directions of visual acceleration and gravity were in the same direction (Fig 3A, 3C and 3E), the stimuli should enhance each other, and the participant would need less visual motion to simulate that they had passed through a given distance compared to when not experiencing a VRI. Contrariwise, when the directions of the visual acceleration and gravity were opposed (Fig 3B, 3D and 3F) the stimuli should oppose each other, and the participant would need more visual motion: the “additive hypothesis”. In this case, the experience of gravity would be the reaction to the downward force of gravity such that when lying supine on a surface, our participants experienced pressure on their backs (or fronts while prone) equivalent to that produced if the bed were accelerating upwards at 9.8m/s/s; in physics this is referred to as the “normal force”.

The task used in this experiment differs from other measures of the sensation of vection [22–25, 31] as it can be completed solely by using optic flow cues (visual motion) [18–21]. Participants do not need to experience a sensation of motion in order to perceive that they have passed through a particular target distance. Instead the distances traveled by the participants in the environment reflect the effectiveness of the optic flow cues in simulating motion of the observer relative to the environment.

In the next two experiments, we varied the likelihood of experiencing a VRI: first by varying the alignment of the available orientation cues, and secondly by varying the orientation cues that were present in the visual environment. In Experiment 2, participants were in the same visual environment as in Experiment 1 where non-gravitational “up” was always towards the top of the head (Fig 2A and 2B). Now, instead of being supine or prone, the participant was always upright with their head tilted forwards in a “prone similar” position (Fig 3D and 3J), or backwards in a “supine similar” posture (Fig 3C and 3I), by close to 90 degrees. In the supine (Fig 3A and 3G) and prone (Fig 3B and 3H) postures used in Experiment 1, the direction of gravity was supported by somatosensory cues, and the internal idiotropic body vector [5] supported the direction indicated by vision. However, when the head was held at 90° to the body (Fig 3I and 3J) there were fewer cues to support the visually indicated direction of “up” and therefore participants should be less likely to experience a VRI. As well, activation of the neck muscles might reduce vestibular ambiguation and support the perception of tilt over the perception of motion [15, 32] referred to as our “head-tilt hypothesis”. Therefore, in experiment 2, when the head is tilted while the body remains in line with gravity and out of line with the visual upright, we expected participants to be less likely to have a VRI or that any VRI they did experience would be less compelling. Our head-tilt hypothesis is that a reduced VRI experience will then result in a reduction in any effect of tilt on the effectiveness of the optic flow cues found when the head is aligned with the body.

In Experiment 3, we varied the visual cues in the environment providing a direction of up. By comparing the effectiveness of an oriented environment (a hallway, Fig 2A and 2B) with one with no orientation cues (a starfield, Fig 2C and 2D) we expected only the oriented environment to be associated with a VRI and therefore gravity to only influence the effectiveness of the optic flow cues in this condition.

## Materials and methods

### Overview

Under various conditions, participants judged how far they had moved within a simulated virtual environment. They were presented with a target at some distance away from them that then disappeared. They then experienced simulated visual motion towards the target’s previous location and indicated when they had reached that location. The distance traveled through the environment was taken as a measure of the effectiveness of the visual motion. The experiments were conducted in agreement with the Declaration of Helsinki and were approved by the ethics committee of York University. All observers signed informed consent forms before taking part in the experiment and were naïve as to the purpose of the study at the time of testing. They had normal or corrected-to-normal vision and reported no vestibular, balance or depth perception problems. Undergraduate volunteers were awarded course credit for their participation.

### Apparatus

In Experiments 1 and 2, stimuli were displayed using the Oculus Rift Developmental Kit 2 (DK2). The DK2 has a field of view that extends approximately 95°(horizontally) x 106° (vertically). The screen has a resolution of 920 x 1080 pixels and a 75hz refresh rate.

In Experiment 3, stimuli were presented in an Oculus Rift CV1 virtual headset. The CV1 has a field of view that extends approximately ±110° diagonally. The screen has a 1,080 x 1,200-pixel resolution per eye and a 90Hz refresh rate. For all three experiments the stimuli were created in Unity (Version 5.5.2f1, Unity Technologies SF, US) on an Alienware Area-51 R2 computer with an intel i7 core and a Nvidia GeForce GTX 980 graphics card. The projection was in stereo and was actively linked to the position of the participant’s head. Therefore, distance cues were available from stereo and parallax.

### Oriented simulated environment: The hallway

The hallway had black and white vertically striped walls (each black or white stripe was approximately 1m wide) and a multicolored stained-glass floor. The simulated width of the hallway was 9m. The walls were 1000m high so the viewer could not see over the top of the walls (see Fig 2B). The walls and the floor extended 10,000m in front of the viewer. There was no ceiling on the virtual hallway so participants could see a blue simulated sky along with a simulated sun that provided the light source. No shadows were added to the environment. There was also a horizon at eye level that participants could see down the hallway and also when the walls were not visible (Fig 2A).

The target used with the hallway was a red 3D rectangular box that was 5m x 1m x 1m in size (see Fig 2A) drawn with one edge towards the viewer. Participants’ eye height was always set to the top of the pillar so that the viewing angle of the scene was the same for all participants. The hallway’s walls were not visible at the same time as the target was presented to stop participants from using them as a marker of distance. The distance to the target was defined as being from the center of the participant (the location of the camera) to the center of the target shape.

### Non-oriented simulated environment: The starfield

The starfield environment (Fig 2C and 2D) was a simulated environment consisting of white spheres and cubes on a black background. The simulated width of each cube was 6m and the circumference of each sphere was 6m. The objects were randomly placed in a starfield that was 250 x 250 x 250m in size. The objects were illuminated from front, top, and bottom of the object Each object blinked on and off with its own pattern (on for between 5-10s; off for between 0-10s). This was done to reduce the likelihood that participants could fixate on one object and use it to track their location. The target was the same as used in the hallway and was aligned with the body with the top of the pillar at eye height. The pillar did not rest on a surface but instead appeared floating in the environment.

### VRI assessment

#### Experiment 1 and 2

The experimenter remained in the room with the participant for the first five trials. The experimenter was there to remind the participant of the instructions during the first trial and to answer any additional questions. They remained until the 5^th^ trial at which point the participant was asked about their experience in the hallway. They were asked if they felt they were:

1. Moving horizontally, this might feel like being upright and moving down a hallway
2. Moving upwards, this might feel like flying towards the sky
3. Moving downwards, this might feel like falling towards the ground

The question was asked for each of the three body postures and the order of the three choices was varied. Participants were also given the option to report some combination for experience. The responses were recorded by the experimenter.

#### Experiment 3

It was later felt that these questions could be improved. For instance, participants could perceive the motion as up or down while their posture reflected a different orientation. A participant who was actually prone might paradoxically feel that they were standing but that the motion simulated falling. Additionally, it was felt that the potential for a mixed experience should be stated more explicitly. Lastly, some participants had difficulty following the options, so the questionnaire was run at the end of each body posture once the participant had removed the HMD. However, the experimenter still remained in the room with the participant until after the 5^th^ trial to ensure the participant understood the instructions.

The questionnaire was read in full after the first part of the experiment with the first body posture. For the 2nd and 3rd body postures, the first paragraph was omitted so that it would be less repetitive for the participant.

“I am going to describe three different ways you could have felt while you were in the virtual environment. It’s important that you think about how you felt while you were in the virtual environment, while you were stationary and while you were moving.

Due to the nature of virtual reality people can have different experiences. All of these are normal, and I am interested in how you felt while you were in the virtual environment. While you were in the virtual environment you might have felt one of three things, or possibly a combination of them. So, did you feel like you were:

1. looking up, and when moving, moving upwards. You might think of this lying down while flying upwards towards the sky

2. looking forwards, and when moving, moving forwards. You might think of this as moving similar to how you regularly move when not in VR, such as standing upright and moving forwards

3. looking down and when moving, moving downwards. You might think of this as lying down and looking over an edge down a cliff or maybe like falling

4. Some combination or other experience”

**Fig 4:**
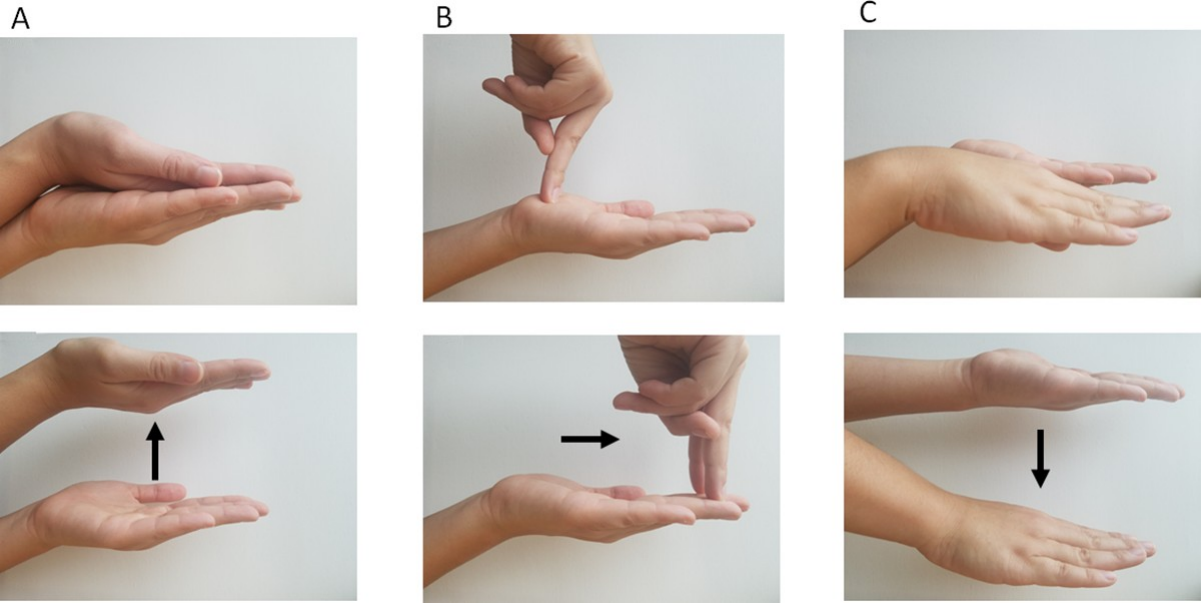
An example of the hand positions used to help clarify the questionnaire. Column A demonstrates the hand posture shown for option 1 in the questionnaire. Column B demonstrates option 2, and Column C demonstrates option 3.

The participant reported which of the four options best described how they felt. The presentation order of options 1–3 were randomized each time they were asked. Option 4 always came last. The participant’s response was recorded, and they were then asked to elaborate on how their body’s orientation felt during the experiment, which was also recorded. Unlike for experiments 1 and 2, this questionnaire was read after completing each posture, instead of during each posture. While this did mean that when answering the questions, the participants had to reflect back on their experience, it allowed for the experimenter to use props to demonstrate the perceived postures, for instance the experimenter oriented their hand in different ways to help clarify the orientation and motion being described (Fig 4).

### Methods specific to Experiment 1

#### Participants for Experiment 1

The experiment had 18 participants (mean age 27.50 ± 7.68yrs, 9 females).

#### Body conditions for Experiment 1

There were three body conditions: (1) an upright viewing condition where the participant stood upright, (2) a supine condition where the participant lay on their back on a massage bed with their feet up against a wall, and (3) a prone condition where the participant lay on their stomach on the massage bed with their head hanging over the edge of the table and the underside of their chin resting on the side of the bed. A box was placed against the soles of their feet during the prone posture to simulate the wall against the feet during the supine posture. In all three body conditions the whole virtual environment was rotated relative to gravity so that the visual display appeared exactly the same, with up corresponding to the top of the head in all conditions.

#### Procedure for Experiment 1

Participants wore the virtual reality headset and were positioned in one of the three body postures. At the beginning of every trial, participants saw the target shape projected at either 10, 20, 40, 60, or 80m sitting on the textured pavement (Fig 2A). Participants were instructed to pay attention to how far away the target was from them and, when ready, to click the left mouse button. Immediately upon the click, the target disappeared, the hallway walls appeared, and the optic flow simulated an acceleration of 9.8m/s^2^ down the hallway. When the participant felt that they had reached the location of the previously visible target (that is, their head was inside where the target had been) they clicked the right mouse button. Once this button was clicked the next trial started with the participant was repositioned at their original position in the hallway, the target appeared at a new distance, and the walls were again rendered invisible.

In each experiment and for each body condition the target distance was chosen pseudo-randomly. The order of the body conditions was determined using a Latin square method. Each target distance was presented to each participant 10 times resulting in 50 trials per participant per body condition. There were two additional trials, one at the beginning of the experiment and one at the end. These were not used in the analysis. The first was used to familiarize the participant with the environment and how the trials worked and in the last trial the target was presented at 200m to indicate the experiment was over.

### Methods specific to Experiment 2

#### Participants for Experiment 2

The experiment had 18 participants (mean age 25.89 ± 4.83yrs, 6 females).

#### Body position for Experiment 2

**Fig 5:**
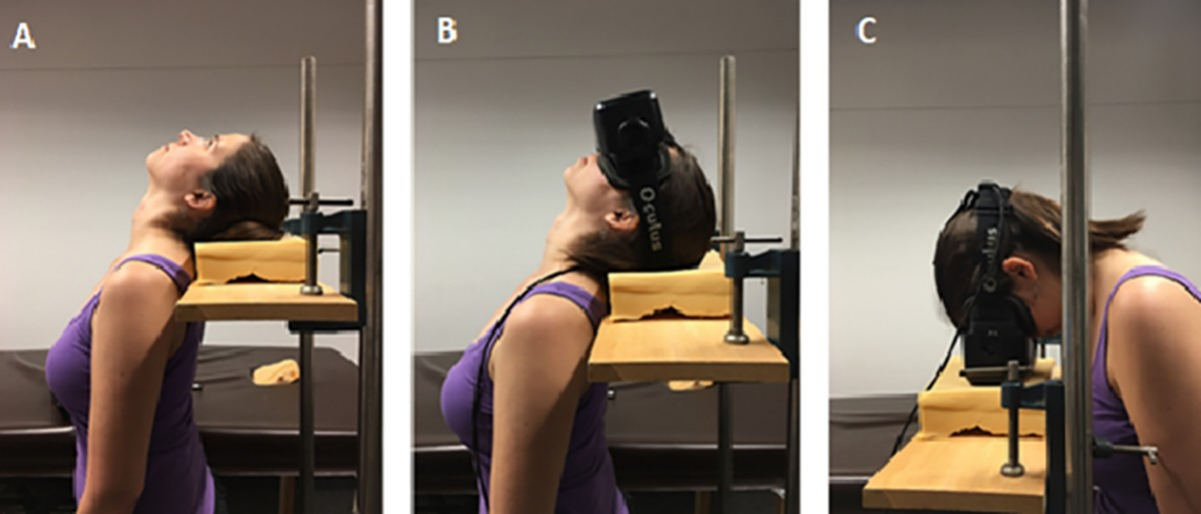
The supine similar and prone similar postures. A demonstrates how the participant’s head was positioned while in the supine-similar position. B shows what this looked like with the oculus on. C shows how the head was positioned during the prone-similar position. In both cases the back was kept as straight as possible. The individual in this manuscript has given written informed consent (as outlined in PLOS consent form) to publish these images.

As in Experiment 1, there were three body postures, but here they were created by bending only the neck (see Fig 5). (1) An upright viewing condition where the participant stood upright. (2) A “head up” condition that mimicked the otolith placement of the Experiment 1 supine condition, referred to as the “supine-similar” posture and (3) a “head down” condition that mimicked the otolith placement of the prone condition in Experiment 1, referred to as the “prone similar” posture. In the head tilt conditions participants rested either the back of their head or the front of the Oculus on a foam pad mounted on an appropriately adjusted wooden plank. Two orthogonal spirit levels were used to ensure the plank was perfectly flat. In the supine-similar posture (Fig 5A) participants were asked to place the back of their head on the foam and to fixate a point directly above them so that their head was approximately a 90° to the floor and their back and shoulders were straight. Once the plank was adjusted to the correct height, participants put the oculus on and were instructed not to raise their head from the plank during the experiment (Fig 5B). In the prone-similar condition participants were asked to stand behind the plank and stare directly down at their toes while their back and shoulders were as straight as possible (Fig 5C). Participants then placed the oculus on their head and the plank was adjusted to approximately the correct height at which point the participants placed the face of the oculus on the foam to test the height to ensure it was correct. This was repeated until the height was correct.

In all three body conditions the whole virtual environment was rotated so that the horizon, the light source and the hallway appeared exactly as in Experiment 1.

#### Procedure for Experiment 2

The procedure and stimuli were the same as in Experiment 1. The differences were only in the body postures.

### Methods specific to Experiment 3

#### Participants for Experiment 3

The experiment consisted of 36 participants (mean age 21.17 ± 4.69yrs, 19 females). Participants were alternately assigned to either the hallway (18 participants, mean age 20.72 ± 4.11yrs, 9 females) or the starfield (18 participants, mean age 21.61 ± 5.28, 10 females) environment, ensuring as even a sex split as possible.

#### Procedure for Experiment 3

The procedure was identical to the other two experiments.

## Data analysis

### Outlier analysis

The same initial outlier analysis was performed in all three experiments.

Firstly, for each person the data was divided into the three separate postures and organized by target distance. Each participant had 150 data points (5 target distances, 10 repetitions, 3 postures). Any mistrials were removed. For instance, a participant could mistakenly skip a trial if they hit the right mouse button twice in a row resulting in a recorded travel distance of less than 1m. Since the acceleration used was 9.8m/s/s a distance of less than one meter would correspond to the participant pressing stop around 1/10^th^ of a second after starting. Then, for that participant and for that target distance, the average and standard deviation (SD) of each distance measure was found. A data point was removed if it was ±2SD away from the mean. Out of the 150 data points per person, on average 7.00 ±2.89 (4.67%) were removed in Exp 1, 6.83 ±1.34 (4.56%) were removed in Exp2, 7.78 ±2.07 (5.19%) were removed in Exp3 Hallway, and 6.67 ±2.49 (4.44%) were removed from Exp 3 Starfield. Then the new averages for each distance were calculated.

In addition to data being removed according to these criteria, participants were also excluded for other reasons. The first was if they were unable to distinguish between the target distances. For example, in experiment 1 one participant was excluded because, while standing in the starfield, they were unable to distinguish between 40m, 60m, and 80m. (mean for 40m = 128.29m, SD = 37.27; 60m = 129m, SD = 22.93; 80m = 126.68m, SD = 42.90). This resulted in one participant being removed from each of these experiments: experiment 1, experiment 3 hallway, and experiment 3 Starfield. In addition to this one further participant was removed in experiment 1 as they kept lifting their head while supine despite being told to keep their head on the surface. One other person was removed from Exp 3 as the screening criteria required that they did not play varsity sports, but they did.

Once these outliers were removed participants in each experiment were collected into their respective datasets.

While testing the assumptions of the ANOVAs that would form the statistical analysis both Exp 3 Hallway and Exp3 Starfield were found to still have significant outliers. This was tested using a boxplot to check for outliers where some data points fell 3 boxes away from the upper and lower hinges (the hinges for the central 50% of the data around the median) indicating the data points fell outside of 99% of the rest of the distribution. This resulted in data points being removed from two participants in experiment 3 Hallway and one person in Exp3 Starfield. Because the ANOVA requires complete datasets to function these three participants were excluded from the analysis. Applying these outlier criteria resulted in 18 participants in each of the experiments and environments.

### Questionnaires

The data from the two questionnaires were analyzed the same way. For all three experiments if a participant responded with a 1 (see questionnaire descriptions above), or a description that best matched a 1, then for that actual body posture, their “perceived posture” was recorded as “felt supine”. If their response was a 2 or best matched a 2, their perceived posture was marked as “felt standing”, and for a response that was a 3 or best matched a 3 was recorded as “felt prone”. For the 4^th^ option, if participants indicated they did not feel like any of the other 3 options, or if their verbal description was a mixture of responses, for instance “I felt like I was standing, but moving downwards”, this was recorded as a “felt other”. The results were then tabulated, see Table 1. Chi-squares were run on the responses from experiment 3 to see if there was a difference in the amount of VRI reported in the starfield vs the hallway, and if the amount of VRI differed between the supine and prone postures.

**Table 1:** Overall VRI rates for the three experiments.

	Posture felt/ actual	Felt Standing	Felt Supine	Felt Prone	Felt Other
Experiment 1 Hallway	Standing	100% (18)	0	0	
	Supine	94% (17)	0	0	6% (1)
	Prone	100% (18)	0	0	0
	Overall VRI Rate:	97%			
Experiment 2 Hallway	Standing	100% (18)	0	0	0
	Supine Similar	83% (15)	6% (1)	0	11% (2)
	Prone Similar	94% (17)	0	0	6% (1)
	Overall VRI Rate:	89%			
Experiment 3 Hallway	Standing	94% (17)	0	0	6% (1)
	Supine	28% (5)	56% (10)	0	17% (3)
	Prone	72% (13)	0	17% (3)	11% (2)
	Overall VRI Rate:	50%			
Experiment 3 Starfield	Standing	83% (15)	17% (3)	0	0
	Supine	28% (5)	44% (8)	0	28% (5)
	Prone	22% (4)	0	78% (14)	6% (1)
	Overall VRI Rate:	34%			

Columns indicate participants’ perceived orientation; rows indicate their actual posture. The numbers in brackets denotes the number of participants who reported that experience in that posture. The percentage is calculated by dividing the number in the brackets by the total number of participants in that experiment. The overall VRI rate is the mean of the supine and prone data.

Data for all experiments were the simulated travel distance necessary for participants to believe that they had reached the position of the previously seen target. The statistical analysis comprised repeated measures analysis of variances (ANOVAs).

## Results

### Variation in required travel distance

**Fig 6:**
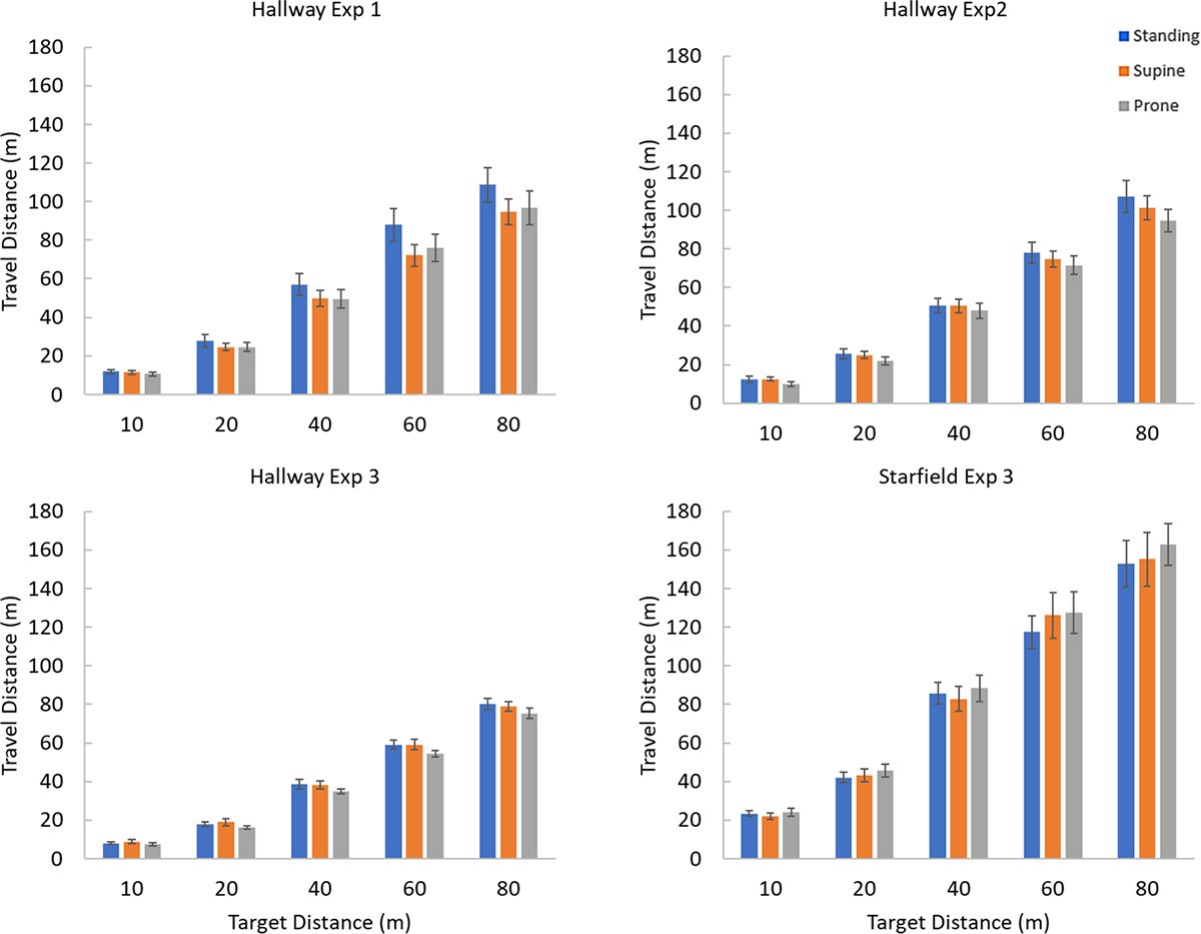
The average simulated travel distance needed to reach each target for each posture for each experiment. Blue bars indicate responses while standing, orange while supine, and grey while prone. Error bars are ± standard error.

The mean distances at which participants indicated that they had arrived at the target position are shown for all conditions and target distances in Fig 6. Pressing the button sooner (shorter travel distances) indicates a more powerful effect of visual motion at simulating motion through the environment as less visual motion was needed for the participant to perceive that they had traveled through the target distance. Four separate 3 (body postures) x 5 (distances) repeated measures ANOVA were performed, one for each experiment. Mauchly’s test of sphericity was used and violations of the sphericity assumption were corrected using the Greenhouse-Geisser correction. Alpha was set at *p* < .05 and post-hoc multiple comparisons were made using least squares difference (LSD).

#### Target distance

Four 3 (body postures) x 5 (distances) repeated measures ANOVA were performed for each experiment. For all experiments and viewing conditions, the target distance influenced the travel distance. Experiment 1 *F*(1.14, 19.45) = 147.88, *p* < 0.001, η^2^_p_ = 0.897, Experiment 2 *F*(1.28, 21.85) = 230.56, *p*< 0.001, η^2^_p_ = 0.931, Experiment 3 (hallway) *F*(1.61, 27.36) = 1092.51, *p<* 0.001, η^2^_p_ = 0.985, and Experiment 3 (starfield) *F*(1.20, 20.36) = 122.58, *p*< 0.001, η^2^_p_ = 0.878. A post hoc pair wise comparison with LSD correction was done for each of the experiments and found that all distances differed from each other (*p*<0.001) which means that participants were successfully able to distinguish the five target distances.

#### Body posture

For Experiment 1, in which whole-body posture was varied, a significant main effect of posture was found *F*(1.68, 28.64) = 7.68, *p* = 0.003, η^2^_p_ = 0.311. A post hoc pairwise comparison with LSD correction of the main effects found that the perceived distance of travel when in the standing position differed from supine position (*p* = 0.006) and the prone position (*p* = 0.009). The supine and prone positions did not differ from each other (*p* = 0.576).

For Experiment 2, in which only head posture was varied to generate supine-similar and prone-similar conditions, a significant main effect of posture was also found *F*(1.76, 30.00) = 4.91, *p* = 0.017, η^2^_p_ = 0.224. A post hoc pairwise comparison with LSD correction of the main effects found that the upright posture differed from the prone-similar posture (*p* = 0.005) but that the supine-similar posture did not differ from the upright posture (*p* = 0.365). The prone-similar and supine-similar postures also differed (*p* = 0.031).

For Experiment 3 (using the hallway) a significant main effect was found for posture *F*(2, 34.00) = 3.85, *p* = 0.031, η^2^_p_ = 0.185. A post hoc pairwise comparison with LSD correction of the main effects found that the prone posture differed from standing (*p* = 0.042) but the prone and supine (*p* = 0.050) and supine and standing did not differ (*p* = 0.877).

For Experiment 3 (using the starfield) no effect was found for posture *F*(2, 34) = 0.68, *p* = 0.51, η^2^_p_ = 0.038.

#### Experiment 3 environment

A mixed 2 (environment) x 3 (body postures) x 5 (distances) ANOVA was performed to explore the effect of environment on travel distance. The between subjects factor (environment) was significant *F*(1, 34) = 69.36, *p*< 0.001, η^2^_p_ = 0.67, where the average distance traveled in the hallway was 39.7m (SE = 3.98) and the starfield was 86.6m (SE = 3.98). There was also a significant interaction between the environment and the distance traveled for each target distance *F*(1.21, 41.12) = 27.78, *p*< 0.001, η^2^_p_ = 0.45. A post hoc analysis with LSD correction found that the distance traveled for each target distance differed based on the environment (*p*< 0.001 for all cases) with participants in the hallway condition needing to travel about half of the distance required in the starfield (Fig 6).

### Variance in VRI likelihood

The frequency of VRI reports is summarized in Table 1 for all three experiments. For Experiment 1 (using the hallway stimulus) all participants reported a VRI except for one person who felt “other” while supine (97%). For Experiment 2 with just head bent, a VRI was experienced 89% of the time. For Experiment 3, 50% had a VRI with the hallway stimulus which was reduced to 34% in the starfield (*X*^2^(1) = 5.31, *p*<0.021). Chi squared analyses showed that in the hallway environment of Experiment 3, the reported VRI experience was significantly affected by posture (*X*^2^(1) = 7.30, *p* = 0.007), with more people reporting VRI while prone (72%) compared to while supine (28%). Posture did not affect VRI responses in the starfield (*X*^2^(1) = 0.966, *p* = 0.326), with 28% of people reporting a VRI while supine and 22% reporting a VRI while prone.

Overall, the hallway environment was much more likely to lead to a VRI compared to a starfield environment. Additionally, in Experiment 3, while in the hallway participants were much more likely to have a VRI while prone compared to supine. No such supine/prone difference occurred in the starfield or the other 2 experiments.

### The variation of gain by experiment and VRI likelihood

The average gain of the travel distances for each condition were calculated and are expressed in Table 2 where gain is defined as perceived distance (the target distance) expressed as a fraction of the actual distance travelled.

**Table 2:** The mean gain for each condition.

	Exp 1 Body Posture	Exp 2 Head Only	Exp 3 Hallway	Exp 3 Starfield
Standing	0.84 (0.07)	0.86 (0.06)	1.15 (0.06)	0.52 (0.03)
Supine	0.91 (0.06)	0.85 (0.05)	1.14 (0.06)	0.53 (0.04)
Prone	0.97 (0.08)	1.00 (0.08)	1.30 (0.10)	0.51 (0.05)

The gain was calculated by dividing the target distance (perceived distance) by the average distance traveled for each target distance (actual distance), and then computing the average across all target distances. This was done for all three postures in each of the three experiments. Note that for Experiment 2, the postures were supine-similar and prone-similar as only the head-on-trunk position was varied. Numbers less than 1 indicate that participants had to travel further than the target distance to feel they had passed through the target distance (low gains) and vice versa. The numbers in brackets are standard errors.

**Fig 7:**
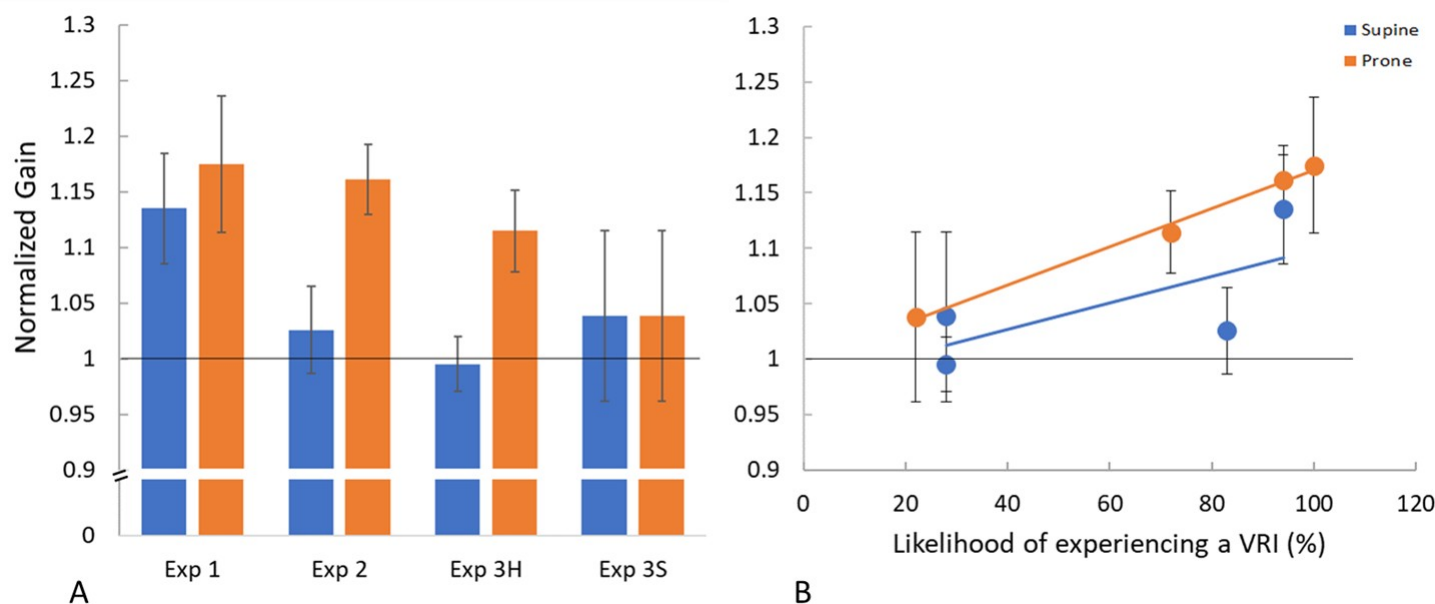
The gain ratio as a function of VRI likelihood. Both figures show the changes in gain while supine and prone relative to when standing. The solid horizontal line at 1 indicates no change with posture. In both figures blue refers to the supine data and orange refers to the prone data. Error bars are ± standard error. A shows the change in gain calculated for each participant in each experiment and environment and then averaged. B shows the same gains relative to standing in A plotted as a function of the likelihood of experiencing a VRI for all three experiments and all environments. The lines are best fit linear regressions.

To assess the change in gain relative to standing for each condition, the gain while supine or prone for each participant, experiment and environment was then expressed as a fraction of the relevant standing gain. The amount of visual motion needed to arrive at a target while standing upright in a particular environment could be thought of as the “standard” or default amount of motion needed to simulate motion through that environment for a given participant. Changes to this amount reflect changes in the effectiveness of the visual motion where numbers greater than one indicate an enhancement of the gain while tilted relative to standing, and numbers less than one indicate a reduction in the gain relative to standing. These ratios were then plotted as a function of the likelihood of experiencing a VRI in that condition (see Table 1) in Fig 7.

### Post hoc analysis of the relationship between VRI rate and gain, posture, and environment

In the results described above we found that in situations where the VRI rate was high the average distance traveled tended to differ from standing. When the VRI rate was low the average distance traveled did not differ from standing. In order to confirm this relationship a Pearson’s correlation (2 tailed) was used to determine the relationship between the gain ratio and VRI rate (Table 1). The relationship between gain ratio and posture (supine relative to standing = 0 or prone relative to standing = 1), and gain ratio and environment (hallway = 0 and starfield = 1) was assessed using a point-biserial correlation (2 tailed) as they are dichotomous categorical variables.

The VRI rate was found to be significantly positively correlated with the gain ratio, though the correlation was small, r(142) = 0.228, p = 0.006 whereas the VRI rate increased so did the gain.

Posture was found to trend toward a significant positive correlation with the gain ratios r_pb_(142) = 0.162, p = 0.052. In agreement with the chi squares above there was also a significant positive correlation between VRI rate and posture, r_pb_(142) = 0.219, p = 0.008, where the prone posture was more likely to lead to a VRI compared to the supine posture. We argue that any correlation between posture and gain ratio is an indirect effect of the VRI rate and posture where the prone posture would have a higher gain due to the higher VRI rate. So we partialed out the effect of VRI in the correlation between posture and gain ratio and the result was no longer significant (r_pb_(141) = 0.118, p>0.05).

The environment and VRI rate were negatively correlated (r_pb_(142) = -0.739, p< 0.001), where the starfield had a lower VRI rate (Fig 7); this is also supported by the chi square analysis. No significant correlation was found between gain ratio and environment (r_pb_(142) = -0.120, p> 0.05). Of course, the environment does lead to differences in a gain as per Table 2 and the ANOVA, just not once the variation found while standing in that environment is removed.

## Discussion

Overall, in all of the three hallway environments participants required *less* visual motion (higher visual gains) to simulate passing through a given target distance while tilted in a hallway environment compared to a standing posture or a starfield environment (Fig 6) but only if they were experiencing a high level of VRI (Fig 7B). The hallway environment led to participants more often reporting a VRI compared to the starfield (50% vs 34%). The prone posture in the hallway environment in particular elicited more of a VRI experience compared to the supine posture (72% vs 28%). This difference in the prone and supine posture’s VRI rate was not seen in the starfield environment (22% vs 28%). Neither the starfield environment nor the supine posture in the hallway led consistently to the experience of a VRI (Table 1).

### When gravity is not where it should be: The additive hypothesis

Our cognitive hypothesis postulated that there would be changes associated with a VRI and our additive hypothesis postulated that there would be a detectable additive effect such that when participants had a VRI and were supine so that the normal force of gravity and visual motion pointed in the same direction they would need less visual motion (higher gain) in order to perceive that they had arrived at a given target location compared to when standing and, vice versa, when prone with a VRI they would require more visual motion (lower gain) compared to standing. In agreement with the cognitive hypothesis we found an effect of a VRI on the effectiveness of the visual motion (Fig 7B) regardless of the direction of tilt. Instead, in conditions that produced a high level of VRI, participants always required less visual motion (higher gains) relative to standing to reach a given target distance compared to when the VRI rate was lower thus disproving our additive hypothesis.

Referring to Fig 7B, there appears to be a relationship between VRI rate and gain relative to standing. As the likelihood of reporting a VRI increased, the effectiveness of the visual motion also increased compared to the standing posture. Both tilted postures in experiment 1 and the prone posture in experiment 2 were associated with high levels of VRI and had correspondingly high gains compared to standing. On the other hand, participants in the starfield environment reported a VRI much less often than those in the hallway environment and the ratio of their gains while tilted compared to standing were close to 1, this was also true of the supine posture in experiment 3 in the hallway environment which also showed a low VRI rate (see Fig 7A).

Referring to our results, the participants’ high-level interpretation of their environment appears to be an important factor in visual self-motion processing, confirming our cognitive hypothesis. However, it appears that the alteration in perceived self-motion is not an additive process in which the acceleration of gravity is added to or subtracted from the visual motion since it did not matter in which direction the participant faced. Instead, it appears to be a weighting effect in which more weight or emphasis is put on vision when gravity is not where it should be: when it is not aligned with the long-axis of a perceptually upright body.

For the starfield displays, which were far less likely to generate a VRI, participants strongly overshot the target (low gain) compared to their performance in the hallway (Fig 6, Table 2). In an unstructured optic flow field with no orientation cues considerably more visual motion was required to simulate moving through a given distance. This is also consistent with our cognitive hypothesis as a low level of reported VRI is associated with less increase in gain relative to standing. But why is the self-motion gain so low in a starfield?

### Oriented vs unoriented environments

The gains while standing when simulating self-motion in the starfield were substantially lower than when in the hallway (0.52 compared to 0.84 or 1.15 in the hallway, Table 2). That is, participants needed about twice as much visual motion in the starfield as they did in the hallway to in order to perceive they had traveled through the same distance. We do not know why this is. In both instances the targets were viewed binocularly and were displayed at the same range of distances. Few studies have used perceived distance traveled, visual odometry or path integration, as their measure of simulated self-motion–a more common method of assessment being a magnitude estimate of the sensation of vection, often normalized to the display being used with instructions such as “estimate the perceived magnitude where your response to this display is 100%”. During physical walking, it seems that non-visual cues dominate in path integration [33–36] although path integration is possible from visual cues alone [19–21] but this is first time that the effect of visual environment on gain has been systematically investigated. We postulate that the lack of oriented structure is responsible for this lesser effectiveness the visual motion in the starfield, but clearly further study is needed. Previous studies that looked at the experience of vection found that polarized environments [37], more colourful environments [38], and the use of a floor surface [39] all lead to enhancements to the perception of vection, which might stem from enhancements to the optic flow cues. It is also possible that the blinking on and off of the stimuli in the environment might have caused a “loss” of some of the optic flow during the movement through our environments. In the context of this paper, an unstructured environment evokes a poorer interpretation of orientation and therefore, according to our cognitive hypothesis in which the interpretation of the environment affects the effectiveness with which visual motion is processed, will result in a reduced effectiveness in simulating self-motion.

Overall, oriented environments increased the incidence of VRIs compared to visual environments without orientation cues (Table 1). In the hallway environment, participants reported over 70% experience of a VRI, with the exception of the supine posture in Experiment 3 hallway, while in the starfield environment people reported a VRI rate of 34%. This finding is expected and is similar to previous findings by Howard et al. [13] using a mirror bed arranged to make an earth-vertical surface behind the viewer appear parallel to the supine viewer. Adding strong orientation cues, such as a ball resting on a shelf or a standing human, however lead to a strong VRI rate of 60%-80% in which the surface was interpreted as an earth-vertical wall and the supine observer felt as if they were actually standing. Unstable perceptions varied between 1–10%. These values are similar to those found in our hallway environments in the three experiments (see Table 1), though the VRI rate found in Experiment 1 and 2 is higher than the rate reported by Howard and colleagues [13]. It is possible that our immersive virtual environment lead to a stronger VRI experience.

The VRI rate we recorded was higher than expected in the starfield condition (Table 1) as VRIs were not expected to occur at all during exposure to this non-oriented display. It is possible that without a clear visually defined direction of up, the participants perceived orientation was dominated by their idiotropic up (“head is up”, Mittelstaedt [5]). However, referring to Table 1 and the chi-squared analyses the starfield environment did evoke a much lower VRI rate then the hallway. We also saw that in the hallway environment, participants needed much less visual motion than they did in the starfield to simulate passing through a given target distance (Fig 6).

Three participants in the starfield environment reported feeling supine while upright (Table 1). This misperception has been reported previously, such as in Allison et al. [40] where, when upright and viewing a room moving in roll, seven of their 35 participants felt that the they were tilted supine and rotating about a vertical axis on at least one trial. This supine-while-upright perception has also been reported by Howard et al. [37].

Interestingly, there was also a large decrease in VRI occurrence reported in Experiment 3 vs Experiment 1 and 2 for the supine posture—from 80%+ down to 28%. Previous studies have shown large individual differences in the experience and duration of VRIs, with some participants reporting the experience every trial, and others only on some trials [14]. It is possible that VRI experiences varied across the experiments.

### Head tilt vs body tilt: The head-tilt hypothesis

Experiment 2 manipulated head and body tilt separately to determine the effects of the body on visual motion processing. When participants are supine or prone in the hallway environment their body- and visually-defined “uprights” are in line with each other but misaligned with the gravity upright. By having participants stand with their head tilted in a supine-similar or prone-similar orientation, their “body up” (at least below the neck) remains in line with gravity but is no longer aligned with the “visual up” (see Fig 3I and 3J). We hypothesised that in this head-tilt-only condition we would see a reduction in the occurrence of a VRI (relative to the truly supine and prone conditions) as perceived orientation became less reliable and the activation of neck muscles might also support the perception of tilt over translation [15]–our head-tilt hypothesis. However, this was not the case (Table 1) and the rate of VRI occurrence was not significantly reduced. It is possible that in experiment 2 while supine the older questionnaire overestimated the likelihood of experiencing a VRI.

If experiencing a VRI was not causing the increased effectiveness of visual motion cues (higher gains) but instead any changes were exclusively due to body posture, we would expect to see posture-related differences while tilted in both the hallway and starfield environments. However, for both tilted postures in the starfield, the ratio between tilted and standing performance was close to 1 indicating the gains while tilted were very similar to standing (See Table 2 and Fig 7B). Table 1 shows that the prone posture is more likely to evoke a VRI experience than the supine posture in the hallway environment. This is the opposite of what was found by Howard and Hu [14] who found that people were more likely to experience a VRI in a supine rather than a prone posture. However, their prone position involved being tied onto a bed and essentially hanging from the straps–a level of discomfort more akin to our supine-similar condition in experiment 2.

### Alternative explanations for changes in self-motion gain

If participants were using other strategies to arrive at the target distances (such as estimating the time needed to reach the targets), we would expect to see no differences between the standing and the tilted body postures in either environment. Neither of these is the case. Instead it appears that the participants’ high-level interpretation of their environment is an important factor for visual self-motion processing.

Another possibility is that lying down could result in a rescaling of the environment such that distances appear closer when supine or prone. Harris and Mander [41] found that supine participants, or participants who felt that they were supine, perceived a rod to be closer than when they were upright suggesting a rescaling of the perceived size of the environment. However, any such rescaling could not explain our results. If environmental cues were compressed, both the travelled motion and the target distance would be compressed, resulting in the same distance estimations as in a room that was perceived as larger. Additionally, undershooting was not found when our participants were tilted in the starfield environment, or when they were less likely to be experiencing a VRI.

### Misinterpreting the vestibular signal: The reweighting hypothesis

So, if we cannot explain our changes in visual gain in terms of timing, the additive hypothesis or rescaling of the room, then we are left with the cognitive hypothesis which predicted a change in the visual gain without specifying which direction it would go in while experiencing a VRI. Here, we show that the gain is increased during a VRI: why might this be?

The presence of a VRI suggests a conflict between the visual up and the gravity up [42] where the participants have resolved the conflict by giving dominance to the visual cues. A conflict does not have a polarity, which might explain why we did not see undershooting while supine and overshooting while prone as hypothesized in the additive hypothesis. We therefore introduce a reweighting hypothesis which states that when a conflict is experienced, visual cues dominate and therefore visual motion becomes more effective in simulating motion through an environment, resulting in higher visual motion gains than when no conflict is experienced.

During a VRI, participants who are tilted report the feeling of being upright, however the vestibular cue is still present. If participants were not interpreting the vestibular cue as tilt and allowing the visual cues telling them they are upright to dominate, then they might interpret the vestibular cue as motion, suggesting a sort of reverse somatogravic (oculogravic) illusion [43, 44]. With an increased perception of motion we would then expect the higher gains that we observed. While tilted, the vestibular system indicates the direction of gravity’s acceleration, as do the body’s other acceleration sensors, such as the kidneys [12, 45]. The presence of acceleration information from both the head and body, along with the addition of visual motion and visual cues indicating they are upright, might more causally lend itself to the interpretation of translation instead of tilt: this hypothesis has been referred to as the otolith-tilt-translation reinterpretation (OTTR) hypothesis [46] and would be the basis for our additive hypothesis. While the study by Crane [15] provides support for this hypothesis—since participants who were tilted backwards perceived some forward motion and participants who were tilted forwards perceived some backward motion—other studies have failed to find supporting evidence [47], including the experiments reported here.

Another scenario is that participants who were tilted and experiencing a visual-vestibular conflict resolved this conflict by ignoring the gravity vector in favour of vision [14]: our reweighting hypothesis. An increased reliance on visual cues would then lead to an unsigned effect in which the gain was increased (more undershooting) in both orientations (supine and prone) relative to standing. Our reweighting hypothesis is that conflict detection may have introduced a weighting effect in which more weight or emphasis was put on vision when gravity is not where it should be: when it is not aligned with the long-axis of a perceptually upright body. No-VRI individuals might then be less sensitive to visual-vestibular conflicts compared to the individuals who report experiencing a VRI. This could stem from a variety of factors such as differences in processing visual or vestibular cues, attending more to proprioceptive information, or a stronger prior for their attending to their body’s orientation compared to VRI individuals. Then, the amount of VRI experienced in an environment would depend on the level of conflict between the visual up and the gravity up in that environment where if there were a clear visual up that differed from the gravity up more participants would experience a VRI. If the visual up were not clearly defined, then less participants would experience a VRI.

### Reweighting?

Tracking how far one has moved during a physical motion with eyes open involves combining potentially redundant information from multiple sources–in particular vision and vestibular signals. In many instances of multisensory integration this is done by averaging the various signals’ estimates after weighting them according to their reliabilities [48, 49].

Fetsch, Turner, DeAngelis and Angelaki [50] found that when the visual vection cue and the vestibular cue were mismatched, people (and monkeys) dynamically reweighted the emphasis put on each cue. It is possible then that instead of interpreting the vestibular cue as motion, participants in the present experiment viewed the visual information as more reliable and therefore weighted it more heavily. When cues to upright are misaligned we see other examples of higher weighting being given to vision in determining upright [51]. For instance, Ward et al. [52] found that as the head and body are rolled, vision becomes more important for determining the direction of gravity. They argue that as the reliability of the vestibular cue decreases, vision is weighted more. This has also been suggested to occur in microgravity environments where in the absence of a gravity cue, astronauts become more sensitive to visual cues, such as cues to visual motion [25, 53].

**Fig 8:**
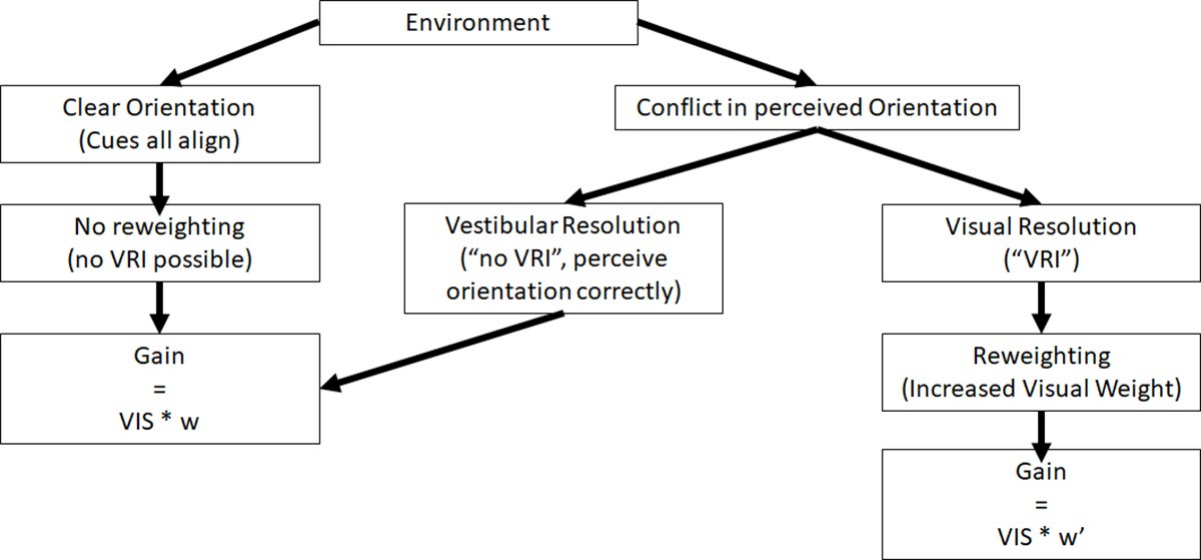
The reweighting model. A model of how the perceived orientation, influenced by visual cues present in an environment, might affect perceived motion. VIS refers the optic flow information. The left side indicates the situation when all cues are aligned. The right side indicates how the detection of conflict may alter perceived travel distance.

In the present experiment participants could either rely on physical cues to tell them they were tilted, or on visual cues to tell them they were standing. A participant who correctly perceived themselves as tilted and experienced visual motion consistent with up or down movement through a tilted visual environment should have no change in their visual weighting compared to the default weight (the visual weight assigned when the person correctly perceives themselves as upright in an upright environment). However, the presence of a VRI would suggest that the person has determined the visual cues are the more reliable for determining upright and therefore weights them higher. A higher visual weight could then lead to changes in the effectiveness of the visual motion cues in simulating movement through the environment, and could explain the undershooting (higher gain) found in this experiment in the hallway environments during a VRI. A model for this is presented in Fig 8. If all cues to upright are aligned, as indicated by the left stream in Fig 8, and the participant correctly feels upright, their visual weighting should be their “default” visual weight (w). The gain of the visual motion would then be processed as the visual information multiplied by w. We postulate that when a visual-vestibular conflict is detected the visual weighting would be increased to w’. In the absence of a detected conflict the gain would be w resulting in performance for no-VRI participants being the same as standing, and but VRI participants having a higher visual weighting (w’) need less visual motion in order to simulate passing through a given distance.

## Conclusion

We have shown that the structure of the environment affects the gain of the response to visual motion. Overall, gains were much higher in a structured environment and depended on whether the observer experienced a VRI (the cognitive hypothesis). As the chances of experiencing a VRI increased so did the effectiveness of the visual motion cue in simulating motion through the environment. Visual effectiveness did not show addition with gravity (the additive hypothesis) but instead was increased when visual-vestibular conflict was resolved by visual domination over vestibular signals that resulted in a visual reorientation illusion (the reweighting hypothesis). Head-tilt did not affect VRI likelihood (the head-tilt hypothesis) presumably because the conflicts it evoked were similar to posture changes of the whole body. These findings suggest that participant’s high-level interpretation of their posture and their environment may lead to changes in visual weighting and a consequent change in the effectiveness of the use of optic flow.
